# Significance of CEA and VEGF as Diagnostic Markers of Colorectal Cancer in Lebanese Patients

**DOI:** 10.2174/1874189400701010001

**Published:** 2007-11-08

**Authors:** Hashem A. Dbouk, Ayman Tawil, Fahd Nasr, Loucine Kandakarjian, Raghida Abou-Merhi

**Affiliations:** 1 Department of Biology, Faculty of Sciences I, Lebanese University, Hadath, Lebanon; 2 Pathology and Laboratory Medicine, American University of Beirut Medical Center, Beirut, Lebanon; 3 Currently at: Department of Biology, Faculty of Arts and Sciences, American University of Beirut, Beirut, Lebanon

**Keywords:** Carcinoembryonic antigen, vascular endothelial growth factor, colorectal cancer

## Abstract

Carcinoembryonic antigen and vascular endothelial growth factors are among the most important prognostic markers of colorectal cancer. Testing for these markers independently has been of limited value in screening for this tumor. The aim of this study is to determine the importance of simultaneous blood CEA and VEGF level determinations in diagnosis of colorectal cancer. Thirty-six patients diagnosed with colorectal cancer along with eight healthy controls were tested by ELISA for CEA and VEGF levels in serum and plasma, respectively. The positive predictive value of these markers was 95.4% for CEA and 89.5% for VEGF, and for combined CEA and VEGF was also high at 88%. Combined CEA and VEGF blood level assay constitutes a useful panel in detecting patients with colorectal cancer. Positive results allow selection of a subgroup of patients with a high tumor risk; therefore, such tests comprise valuable tumor diagnostic tests to add to current detection methods.

## INTRODUCTION

Colorectal cancer is the second most prevalent cancer and the fourth leading cause of death [1]. In Lebanon, it is the second most frequently diagnosed cancer in women and the fourth most frequent cancer in men; with a yearly incidence of about 12 cases per 100,000 individuals [2, 3]. The literature on colorectal cancer in Lebanon is limited and mostly related to its epidemiology. The tumor tends to be diagnosed in its late stages, which underscores the need for more effective detection methods [4–6].

Advances in molecular medicine, specifically in the areas of gene expression and proteomics have made it possible to correlate disease stages with molecular marker profiles. In the case of colorectal cancer, a wide variety of molecular markers have been studied. These studies have mainly focused on carcinoembryonic antigen (CEA), and more recently on vascular endothelial growth factor (VEGF), both of which are found in body fluids including serum and plasma [7–9].

Carcinoembryonic antigen (CEA) is a glycoprotein with a molecular weight of approximately 200 kDa [10, 11]. This antigen is expressed in a number of normal tissues including colon, stomach, tongue, esophagus, cervix, sweat glands, and the prostate [11]. In colorectal cancer however, its concentration is increased and the general distribution of the molecule on the cell surface is also altered. CEA is considered as one of the most clinically significant tumor markers for colorec-tal cancer, providing information on prognosis, tumor recurrence, and metastasis [10, 11].

Vascular endothelial growth factor (VEGF) is a dimeric glycoprotein of 34–42 kDa. It is expressed by a variety of normal cells, but is significantly over-expressed by malignant tumors (such as colorectal adenocarcinoma), where it can be produced by the tumor cells themselves or by stromal cells [12, 13]. This glycoprotein plays a crucial role in many pathologic conditions and malignancies because it stimulates capillary tube formation, endothelial cell proliferation, tumor invasion and metastasis, thereby playing an essential angio-genic role pivotal for tumor growth and aggressiveness [13–15].

The aim of this study is to measure the levels of CEA and VEGF in serum and plasma respectively in order to determine whether they can play a role in detection and diagnosis of colorectal cancer in the Lebanese population.

## MATERIALS AND METHODOLOGY

Our study consisted of patients and controls, both of which are representatives of the Lebanese population with its cultural and religious diversity. The first group comprised of thirty-six patients with colorectal carcinoma (20 males and 16 females, mean age 67 years, age range 24 to 90 years) diagnosed at the American University of Beirut Medical Center (AUBMC) between October 2004 through June 2007. The control group consisted of eight healthy volunteers (6 males and 2 females, mean age 40 years; range 25 to 61) that were studied during the same period. Serum and plasma from the patients and controls were obtained and evaluated for CEA and VEGF levels.

At the time of enrollment in the study, 7 to 10 milliliters of whole blood were drawn from the anticubital vein from each patient preoperatively, and from each control individual after clinical evaluation. Blood samples were drawn separately into vacutainers containing sodium ethylenediamine tetra-acetic acid (EDTA-Na) for plasma and non EDTA-Na vials for serum. The samples were then centrifuged at 3500 rpm for 3 minutes (directly after extraction for plasma, and after waiting at room temperature for 20 to 30 minutes for serum), and then stored at −80° C until analyzed.

CEA serum concentration was determined by quantitative enzyme-linked immunosorbent assay (ELISA) (IBL CEA ELISA, Immuno-biological laboratories, Hamburg, Germany). VEGF plasma concentration was also determined by quantitative ELISA (Quantikine, R&D Systems, Minnea-polis, USA). All samples were run in duplicate. The experiments were repeated three times and calculations were based on mean values in order to ensure accuracy.

Plasma samples instead of serum samples were used for VEGF levels in order to avoid inaccuracy resulting from the well known production of VEGF by platelets, thereby making the results more reliable and reproducible [16].

Statistical analysis was performed by Student’s t-test for independent samples using SPSS 15.0 for Windows statistical software, with α < 0.05 considered as statistically significant. Analysis included the following variables: CEA and VEGF levels, patient gender, age, tumor location, lymph node involvement, and presence of distant metastasis. Serum levels of CEA and plasma levels of VEGF were considered as pathological when they exceeded the mean plus two standard deviations of the control groups.

## RESULTS

Patient characteristics are summarized in Table 1. CEA levels were assayed in all 36 patients, whereas VEGF levels were assayed in 27 of the 36 patients. The relatively small sample size is a reflection of the incidence rates of cancers in Lebanon, due to the small population size, as well as the low detection frequencies as many cancers go un-noticed or are diagnosed at terminal stages. Therefore, this provides direct evidence for the need to devise easy and cheap methods for cancer detection in the Lebanese population.

The cut-off values for both CEA and VEGF were calculated as the mean values obtained from the control cases plus 2 standard deviations. CEA levels < 3.8 ng/ml and VEGF levels < 12.84 ng/ml were considered normal.

**Fig. (1):**
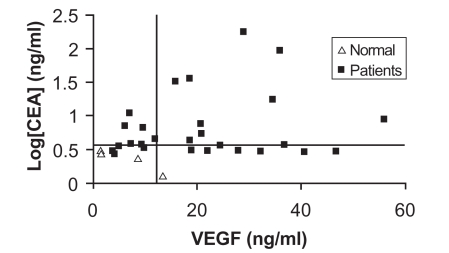
Patient and normal control distribution depending on both CEA and VEGF levels: The patient distribution, for most patients, is clearly distinct from the normal samples for both CEA and VEGF. The horizontal line represents the CEA cut-off level at 3.8 ng/ml (log[CEA] = 0.58), while the vertical line is the VEGF cut-off level of 12.84 ng/ml.

The median CEA levels in patients and controls were 15.13 ng/ml (range 2.53 to 176.51 ng/ml) and 2.52 ng/ml (range 1.31 to 3.218 ng/ml), respectively. The median VEGF levels for patients and controls were 21.22 ng/ml (range 3.82 to 55.82 ng/ml) and 4.52 ng/ml (range 1.375 to 9.61 ng/ml) respectively (Fig. 1). Forty-eight percent of the patients had lymph node metastasis and 50% of the patients had distant metastasis. Both metastatic groups had CEA and VEGF levels that were higher than those of controls, but this difference was not statistically significant (p > 0.05). With respect to tumor differentiation, CEA and VEGF levels were near the cutoff levels in well-differentiated tumors, and much higher in moderately to poorly differentiated tumors, however, we were unable to include this in our study due to very limited number of patients with well-differentiated tumors (2 of 30) compared to moderate-poorly differentiated tumors (28 of 30), thereby preventing statistical comparison.

Using the cut-off levels mentioned above, specificity was 85.7% for CEA and 71.4% for VEGF, while sensitivity was 58.3% for CEA and 63% for VEGF. Independently, both markers had a high positive predictive value of 95.4% and 89.5% for CEA and VEGF respectively, and when combined, their positive predictive was also relatively high at 88%. The negative predictive value of these two markers was low, 28.5% for CEA and 33.3% for VEGF. The combination of both markers (using our cut-off values and those used in other studies) also gave a very low negative predictive value at 28% (Table 2).

## DISCUSSION

Among the prognostic and diagnostic markers studied in colorectal cancer, the most significant are CEA and VEGF. They are linked to tumor growth and metastasis as well as other tumor properties [8, 11, 14]. VEGF plays a key role in angiogenesis, a highly complex process that is essential for tumor growth. Angiogenesis is regulated by various pro- and anti-angiogenic factors. One of the most important pro-angiogenic factors is VEGF, which is secreted by a variety of tumors and is regulated by the tumor cells themselves as well as by the tumor microenvironment [12, 14]. CEA, on the other hand, plays an important role in tumor metastasis, especially to the liver, where it mediates tumor cell adhesion to new sites [11].

Determining the relationship between these two and colorectal cancer, has been the focus of several studies. The results were variable in terms of their role in this tumor as either diagnostic or prognostic markers. Schiemann and coworkers showed higher levels of CEA in hereditary non-polyposis colorectal cancer, and lower levels in sporadic colorectal cancer [18]. A review of various studies concludes that CEA has little diagnostic value; rather it has significant prognostic value in determining patient outcome following surgery or chemotherapy [10]. Still other studies show that the prognostic role of CEA in colorectal cancer may be enhanced with simultaneous use of other markers [18–20]. A recent study by Ferroni and coworkers showed that the use of tissue CEA and VEGF levels is potentially useful for the prognosis of colorectal cancer patients [21].

Studies have also produced conflicting results concerning VEGF as a diagnostic marker in colorectal cancer. Broll and coworkers demonstrated that VEGF has a low sensitivity of 36% [15], whereas our study shows a higher VEGF sensitivity (63%). Other studies showed that VEGF has a significant prognostic role by affecting the tumor’s metastatic potential, and by correlating with response to treatment and survival [16, 22, 23]. Preoperative and postoperative VEGF levels were also shown to correlate with prognosis [24]. Still other studies showed that the combination of CEA and VEGF increases the sensitivity in detecting colorectal cancer [15, 21, 25].

This study compares the significance of CEA and VEGF blood levels, both independently and in combination, as diagnostic markers for colorectal cancer. We found VEGF levels to be much higher in our patient group than in controls when compared to CEA. Most patient CEA levels, in contrast, clustered relatively close to the normal cut-off value of 3.8 ng/ml (or log[CEA] = 0.58) (Fig. 1). Furthermore, our findings differ from those of other studies, which demonstrate that the combination of both markers increases their positive predictive value. In our study, the use of individual markers gave a higher positive predictive value than the combination of the two markers at the selected cutoff limits. Lowering the CEA cut-off value to the 2.5 ng/ml figure used in a multitude of other studies as the cut-off value of preference for CEA, while maintaining our VEGF cut-off value of 12.84 ng/ml, increased the combined positive predictive value from that observed with our cutoffs (88%) to 89.5%. The low negative predictive values of these two markers in the Lebanese population indicates that results from such test should serve only as preliminary tests, and should be followed by others in case of suspicion of cancer or family history.

## CONCLUSION

This study indicates that the combination of serum CEA and plasma VEGF levels, interpreted with the cut-off levels used here, appears to have value in the detection of colorectal cancer in our group of patients, and potentially for Lebanese and other patients. This is by no means a specific test since CEA itself may be detected in a variety of tumors such as lung and breast carcinoma, among others [11]. We conclude, based on the lack of statistically significant diagnostic value from our study, and as a conclusion of various studies in the literature, that the main value of these two markers still lies in the fact that they provide greater insight into prognosis of colorectal cancer. Even though our study did not show this, the lack of prognostic value can be traced to the small patient number not to absence of prognostic correlation, as values between controls and patients, as well as between the different classifications of patients, were clearly different in observation, but not so statistically as overall.

If CEA and VEGF levels are to be used for diagnostic purposes, they should be coupled with colonoscopy and biopsy, which remain the golden standards in detection and diagnosis of colorectal carcinoma, and possibly with various stool screening approaches such as fecal occult blood and other potential stool markers [26]. Concomitant high CEA and VEGF levels can potentially select out a subgroup of individuals who may require either earlier or more frequent screening colonoscopy. Until the time comes when there will be one specific marker for colorectal cancer, additional molecular studies on a larger number of patients are needed to validate the above results.

## ETHICAL APPROVAL

This article conforms to the ethical standards required by the Lebanese University, and which were followed at all stages of the study.

## Figures and Tables

**Table 1.** Significance of CEA and VEGF Values of Patients According to Tumor Characteristics

Features	Patients with CEA	CEA (ng/ml) Mean	p value	Patients with VEGF	VEGF (ng/ml) Mean	p value
Normal Group	8	3.8	-	8	12.84	-
Sex						
Male	20	20.39	0.286	17	17.34	0.059
Female	16	8.67		10	27.84	
Age						
>55	31	15.74	0.774	24	21.36	0.889
≤55	4	10.71		3	20.13	
Tumor Location						
Colon	23	18.59	0.357	17	20.1	0.821
Rectum	12	7.67		10	21.38	
Distant Metastasis						
Yes	17	22.82	0.184	14	23.6	0.372
No	17	7.56		13	18.67	
Lymph node involvement						
Yes	14	25.52	0.151	13	22.77	0.469
No	15	6.23		12	18.49	

**Table 2.** Evaluation of Diagnostic Value of CEA and VEGF

	CEA (3.8 ng/ml)	VEGF (12.84 ng/ml)	CEA & VEGF (3.8 ng/ml; 12.84 ng/ml)
Specificity	85.7%	71.4%	81.4%
Sensitivity	58.3%	63%	66.67%
Positive predictive value	95.4%	89.5%	88%
Negative predictive value	28.5%	33.3%	28%

## References

[1] Parkin DM, Bray F, Ferlay J, Pisani P (2005). Global Cancer statistics 2002. CA Cancer J Clin Cancer.

[2] Shamseddine A, Sibai AM, Gehchan N (2004). for The Lebanese Cancer Epidemiology Group. Cancer incidence in postwar Leba-non: Findings from the first national population-based registry, 1998.. Ann Epidemiol.

[3] Adib SM, Mufarrij AA, Shamseddine AI, Kahwaji SG, Issa P, El-Saghir NS (1998). Cancer in Lebanon: an epidemiological review of the American University of Beirut Medical Center tumor registry (1983-1994). Ann Epidemiol.

[4] Ibrahim NK, Abdul-Karim FW (1986). Colorectal adenocarcinoma in young Lebanese adults. Cancer.

[5] Nabbout G, Hajjar L, Khoury G (1992). Rectal cancer: 10 years experience at AUB-MC. J Med Liban.

[6] Daher M (1998). Colorectal cancer: prevention, screening and management. J Med Liban.

[7] Reymond MA, Steinert R, Kahne T, Sagynaliev E, Allal AS, Lip-pert H (2004). Expression and functional proteomics studies in colorectal cancer. Pathol Res Pract.

[8] Kahlenberg MS, Sullivan JM, Witmer DD, Petrelli NJ (2003). Molecular prognostics in colorectal cancer. Surg Oncol.

[9] Hamilton SR (1999). Colon cancer testing and screening. Arch Pathol Lab Med.

[10] Duffy MJ (2001). Carcinoembryonic antigen as a marker for colorectal caner: Is it clinically useful?. Clin Chem.

[11] Hammarstrom S (1999). The carcinoembryonic antigen (CEA) family: structures, suggested functions and expression in normal and malignant tissues. Semin Cancer Biol.

[12] Cascinu S, Staccioli MP, Gasparini G (2000). Expression of vascular endothelial growth factor can predict event-free survival in stage II colon cancer. Clin Cancer Res.

[13] Zebrowski BK, Yano S, Liu W (1999). Vascular endothelial growth factor levels and induction of permeability in malignant pleural effusions. Clin Cancer Res.

[14] Hicklin DJ, Ellis LM (2006). Role of the vascular endothelial growth factor pathway in tumor growth and angiogenesis. J Clin Oncol.

[15] Broll R, Erdmann H, Duchrow M (2001). Vascular endothelial growth factor (VEGF) – a valuable serum tumour marker in patients with colorectal cancer?. Eur J Surg Oncol.

[16] Hyodo I, Doi T, Endo H (1998). Clinical significance of plasma vascular endothelial growth factor in gastrointestinal cancer. Eur J Cancer.

[17] Schiemann U, Gunther S, Gross M (2005). Preoperative serum levels of the carcinoembryonic antigen in hereditary non-polyposis colo-rectal cancer compared to levels in sporadic colorectal cancer. Cancer Detect Prev.

[18] Fernandes LC, Kim SB, Matos D (2005). Cytokeratins and carcinoembry-onic antigen in diagnosis, staging and prognosis of colorectal ade-nocarcinoma. World J Gastroenterol.

[19] Chen CC, Yang SH, Lin JK (2005). Is it reasonable to add preopera-tive serum level of CEA and CA19-9 to staging for colorectal cancer?. J Surg Res.

[20] Zieglschmid V, Hollmann C, Mannel J (2007). Tumor-associated gene expression in disseminated tumor cells correlates with disease progression and tumor stage in colorectal cancer. Anticancer Res.

[21] Ferroni P, Palmirotta R, Spila A (2006). Prognostic Value of Carcinoembryonic Antigen and Vascular Endothelial Growth Factor Tumor Tissue Content in Colorectal Cancer. Oncology.

[22] Akbulut H, Altuntas F, Akbulut KG (2002). Prognostic role of serum vascular endothelial growth factor, basic fibroblast growth factor and nitric oxide in patients with colorectal carcinoma. Cytokine.

[23] De Vita F, Orditura M, Lieto E (2004). Elevated perioperative serum vascular endothelial growth factor levels in patients with colon carcinoma. Cancer.

[24] Werther K, Sorensen S, Christensen IJ, Nielsen HJ, the Danish RANX05 Colorectal Cancer Study Group (2003). Circulating vascular en-dothelial growth factor six months after primary surgery as a prognostic marker in patients with colorectal cancer. Acta Oncol.

[25] Celen O, Kahraman I, Yildirim E, Berberoglu U (2004). Correlation of vascular endothelial growth factor (VEGF) and CEA with clinico-pathological variables in colorectal cancer patients. Neoplasma.

[26] Osborn NK, Ahlquist DA (2005). Stool screening for colorectal cancer: molecular approaches. Gastroenterology.

